# Arsenic Pollution and Anaerobic Arsenic Metabolizing Bacteria in Lake Van, the World’s Largest Soda Lake

**DOI:** 10.3390/life12111900

**Published:** 2022-11-16

**Authors:** Esra Ersoy Omeroglu, Mert Sudagidan, Erdal Ogun

**Affiliations:** 1 Basic and Industrial Microbiology Section, Biology Department, Faculty of Science, Ege University, Bornova-Izmir 35100, Turkey; 2 KIT-ARGEM R&D Center, Konya Food and Agriculture University, Meram-Konya 42080, Turkey; 3 Molecular Biology and Genetic Department, Faculty of Science, Van Yuzuncu Yil University, Van 65080, Turkey

**Keywords:** Lake Van, arsenic pollution, arsenate, arsenite, anaerobic bacteria

## Abstract

Arsenic is responsible for water pollution in many places around the world and presents a serious health risk for people. Lake Van is the world’s largest soda lake, and there are no studies on seasonal arsenic pollution and arsenic-resistant bacteria. We aimed to determine the amount of arsenic in the lake water and sediment, to isolate arsenic-metabolizing anaerobic bacteria and their identification, and determination of arsenic metabolism. Sampling was done from 7.5 m to represent the four seasons. Metal contents were determined by using ICP-MS. Pure cultures were obtained using the Hungate technique. Growth characteristics of the strains were determined at different conditions as well as at arsenate and arsenite concentrations. Molecular studies were also carried out for various resistance genes. Our results showed that Lake Van’s total arsenic amount changes seasonally. As a result of 16S rRNA sequencing, it was determined that the isolates were members of 8 genera with *ars*C resistance genes. In conclusion, to sustain water resources, it is necessary to prevent chemical and microorganism-based pollution. It is thought that the arsenic-resistant bacteria obtained as a result of this study will contribute to the solution of environmental arsenic pollution problems, as they are the first data and provide the necessary basic data for the bioremediation studies of arsenic from contaminated environmental habitats. At the same time, the first data that will contribute to the creation of the seasonal arsenic map of Lake Van are obtained.

## 1. Introduction

Microbiota members in alkaline habitats such as soda lakes involve anaerobic microorganisms capable of reducing certain metals and metalloids. During the last 20 years, respiratory reduction of selenate [Se(VI)], selenite [Se(IV)], arsenate [As(V)], Fe(III), Co(III), and Cr(VI) by various microorganisms and this process has been shown to be of geochemical, ecological and environmental importance [1].

Among these metalloids, arsenic is a common element that can be found in the environment in different forms (commonly trivalent [As(III)] or pentavalent [As(IV)] form). Inorganic arsenic is the most abundant arsenic species in nature and is commonly found in different environments [2]. It is generally found in soil as insoluble sulfo salts and sulfides such as Arsenopyrite, Orpiment, Realgar, Lollingite, and Tennantite. Although arsenic is naturally found in the earth’s crust, arsenic contamination is mainly caused by various anthropogenic activities such as excessive use of arsenic in pesticides, herbicides, wood preservatives, and medicinal products [3].

Although arsenic is present in living systems at a rate of 2 mg/kg and is called an ‘essential toxin’ because it is required in trace amounts for growth activities [4]. This metalloid is highly toxic to living organisms at high concentrations, and its toxicity is mainly based on its chemical diversity [2]. Although As(V) and As(III) arsenic forms are the most common forms in natural environments, arsine (-III) and elemental arsenic (0) forms are also basically found in nature. Although both arsenate and arsenite are very toxic, the toxicity of arsenite is approximately 100 higher than that of arsenate [3,5]. These two forms induce various types of cellular damage in biological systems. Considering its general mechanism, it is seen that arsenate and inorganic phosphate are structurally very similar. Therefore, both are taken into the cell by the same system. Phosphate is transported across the cell membrane, and by replacing phosphate with arsenic, reactions that require phosphorylation are damaged, and adenosine triphosphate synthesis is inhibited [3]. In bacteria, yeast, and mammals, under neutral conditions, arsenite is taken into the cell by aquaglyceroporins, which are glycerol transport proteins. Arsenite toxicity is due to the ability of cysteine residues in proteins to bind to sulfhydryl groups and, as a result, to inactivate these proteins [5]. Various toxic effects on human health occur when exposure to arsenic is above the concentration allowed by standards for drinking water’s recommended limit of 10 µg/L as set out in the guidelines of the World Health Organization (WHO) and the Environmental Protection Agency (EPA).

From the point of view of microorganisms, it is seen that some microorganisms can cope with arsenic toxicity by using different ways (precipitation, chelation, compartmentalization, extrusion, or biochemical transformation). Therefore, such microorganisms play an important role in the arsenic geocycle [2].

The presence of arsenic in nature is critical to the health of millions of people around the world [5]. Contamination of water and soil with arsenic is a serious threat to public health and the environment worldwide [6]. Because arsenic is highly soluble, it is very difficult to remove arsenic from contaminated water. Looking at the world, millions of people are affected by arsenic in India, Bangladesh, Nepal, Thailand, China, Taiwan, Vietnam, Chile, Hungary, and some parts of the USA [7,8]. In Bangladesh, 57 million people were exposed to arsenic due to the use of arsenic-contaminated wells [9]. When we look at the countries with an arsenic problem, we see that isolation and identification of arsenic-resistant bacteria are carried out by sampling from arsenic-rich environments to solve this problem. Because bioremediation of arsenic in contaminated areas requires arsenic reduction and oxidation using arsenic-resistant microorganisms. For this process to be successful, it is necessary to determine the arsenic metabolism of these microorganisms. For this reason, various studies have been carried out by expert researchers in different countries [9,10,11,12,13,14,15].

When the studies in the literature are examined, it is revealed that regions with high salt content, alkaline pH properties, volcanic and geothermal activity, and anthropogenic factors are preferred for the isolation of arsenic-resistant bacteria [8]. Lake Van has these features. Lake Van is localized on the Eastern Anatolian high plateau in southeast Turkey and has a maximum depth of 445 m. In terms of volume, it is the fourth-largest terminal lake and the largest soda lake in the world [16,17,18]. Due to tectonic movements, there may be a 130 km expansion in the size of the lakes. Nemrut (3050 m above sea level) and Süphan (3800 m above sea level) are semi-active volcanoes located around Lake Van. It is stated that the last eruption took place in Nemrut in 1441. The lake water is salty (21.4‰) and alkaline (155 mEq^−^^1^, pH 9.81) because volcanic rocks undergo chemical erosion and go through the evaporation process [19].

There is no study in which Lake Van was selected as the sampling point for arsenic-resistant bacteria isolation. Therefore, this study was carried out mainly to achieve four main objectives: 1. to make seasonal water and sediment sampling, 2. to contribute to the creation of the arsenic map of Lake Van by revealing the degree of influence of arsenic concentration in this region by seasonal factors, 3. to isolation and identification of anaerobically arsenate-reducing and arsenite-oxidizing bacteria and 4. to determine their arsenic metabolic pathways. In this way, it is thought that it will contribute to the sustainability of Lake Van and all living creatures there.

## 2. Materials and Methods

### 2.1. Study Site

**Figure 1:**
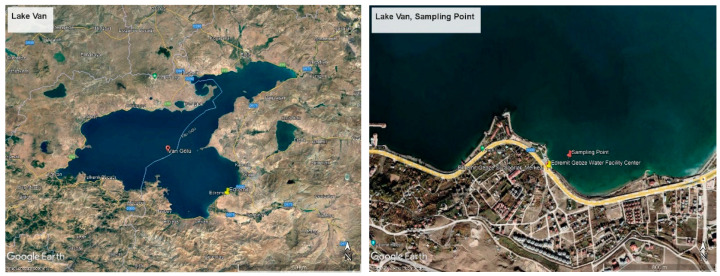
Satellite image of the sampling area.

Lake Van is a volcanic barrier lake formed as a result of the explosion of the Nemrut volcanic mountain, closing the front of the tectonic depression area in the region. The lake is around 430 km from the land. The average depth of the lake is 171 m, and its deepest point is 451 m. The age of the lake is thought to be 600,000 years. The surface area of Lake Van, which has many coves, is 3713 km^2^. Lake Van is an aquatic ecosystem different from both freshwater and marine ecosystems. Besides being the largest soda lake in the world, Lake Van is the fourth largest hydrologically closed lake. The eastern part of Lake Van is a region where tectonic movements are intense due to volcanic activity [20], and this type of mobility is one of the factors that cause arsenic transition to the environment. In addition, anthropogenic pollution agents also cause an increase in the amount of arsenic in the lake. A total of 70 percent of the sewage water belonging to various settlements around the lake is transferred to the lake without treatment, causing the pollution rate to increase gradually. It is estimated that 1800 L of sewage flow into the lake every second. On the other hand, tons of garbage and domestic waste feed the lake continuously in terms of pollution via 18 rivers and streams connecting to the lake. In addition, the existence of various social facilities, the use of which increases in certain seasons, is also a pollution factor. When evaluated in terms of the specified criteria, the Edremit-Gebze water sports facility was determined as the sampling point (Figure 1). The Edremit region is located on the eastern wing of Lake Van and is also a region with a high potential for anthropogenic pollution due to various water sports activities and the presence of various businesses in its vicinity. As an indicator, Aydın et al. have shown some regions are not suitable for microbiological criteria in the Van-Edremit region [21]. In addition, since the earthquake, one of the factors that increase the presence of arsenic occurred in Van on 23 October 2011, it appears as another important factor increasing arsenic pollution. When evaluated in terms of all these factors, as a preliminary study, samples were taken from Edremit, Iskele, Gevaş, Akdamar, and Ergil, and their arsenic contents were analyzed. The highest value and microbial activity were obtained in the Edremit region. For this reason, the Edremit region was chosen as the sampling area.

### 2.2. Water and Sediment Sampling

The amount of water in terminal deep lakes such as Lake Van may vary seasonally [22,23]. Evaporation occurs with increasing temperature, and accordingly, the arsenic amount in lake water may be variable. So, water and sediment sampling representing four seasons from 7.5 m in the Edremit region was performed. Seasonal sampling was carried out from the same coordinate and depth by determining the days when the weather conditions were suitable. This sample does not represent the entire lake. However, since it was determined as the region with the highest arsenic pollution as a result of the preliminary study sampling was carried out in this region to determine how this anthropogenic pollution changes seasonally.

**Figure A1:**
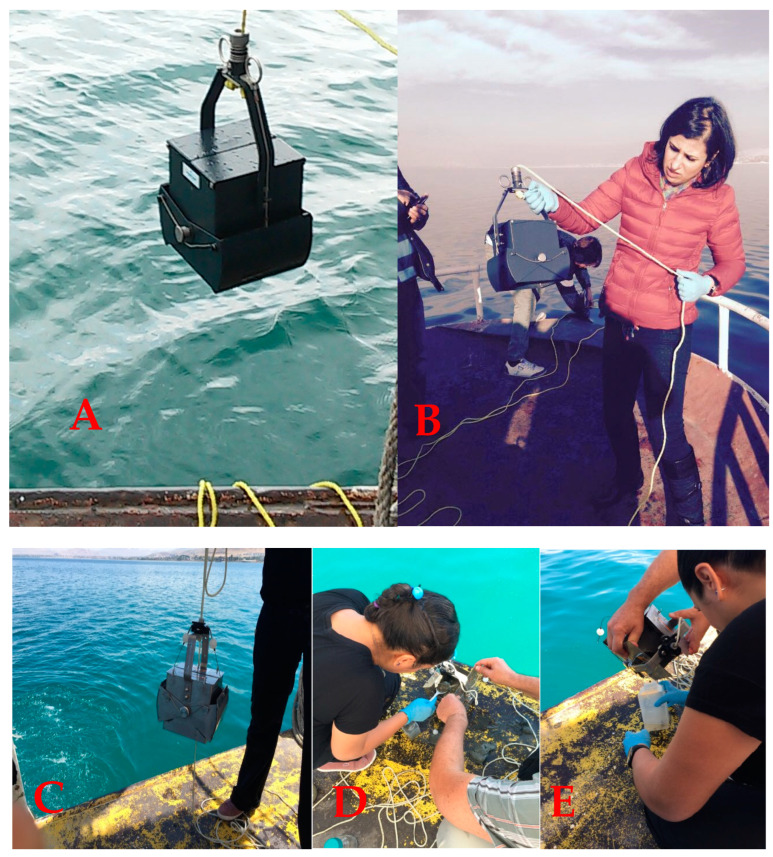

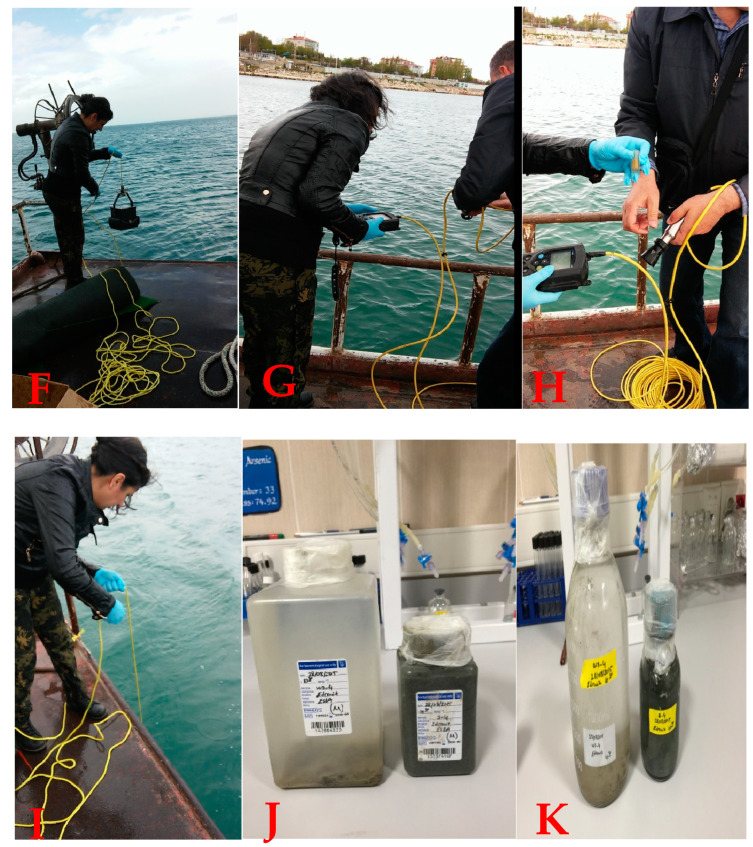
Pressure and salt resistant ladle (**A**–**C**) and sampling (**D**–**K**).

During sampling, the coordinates and various physical parameters, such as pH, temperature, and dissolved oxygen of the sampling point, were determined (see Appendix A Figure A1). Sampling codes and designated physical parameters are seen in Table 1.

### 2.3. Chemical Analysis

Water and sediment samples collected seasonally from the sampling point at Lake Van were analyzed for total arsenic analysis using Inductively Coupled Plasma Mass Spectrometry (ICP-MS) [24]. The samples were examined in terms of 26 different heavy metals. All heavy metal analyzes were performed using the Epa Methods 3051 analysis method [25].

### 2.4. Isolation of Anaerobic and Alkaliphilic Arsenate-Reducing and Arsenite-Oxidizing Bacteria

**Figure A2:**
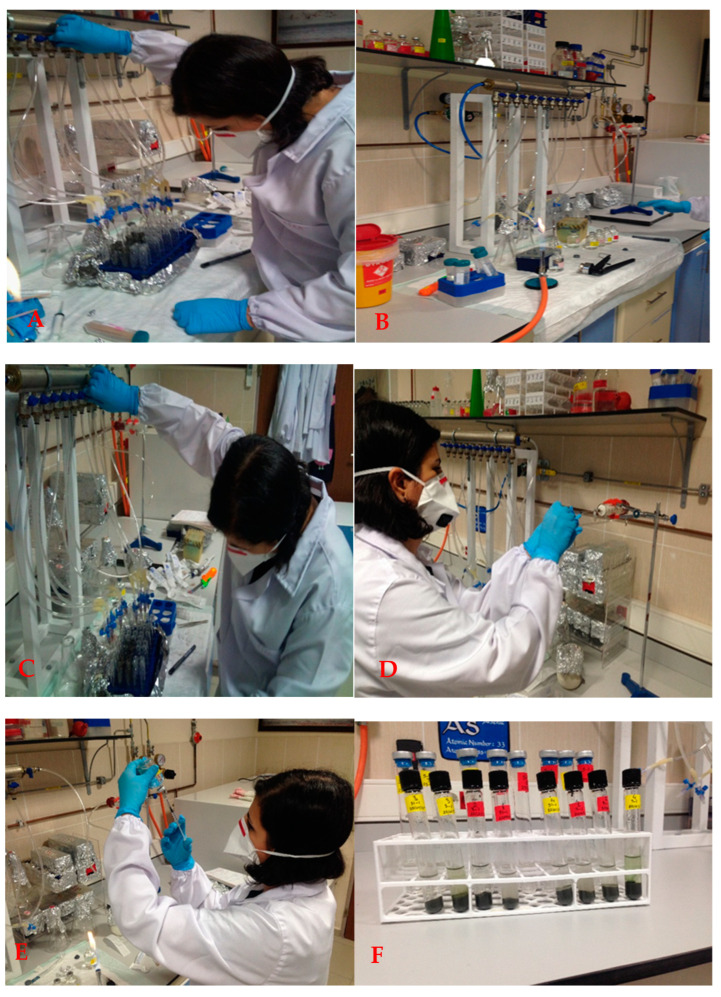

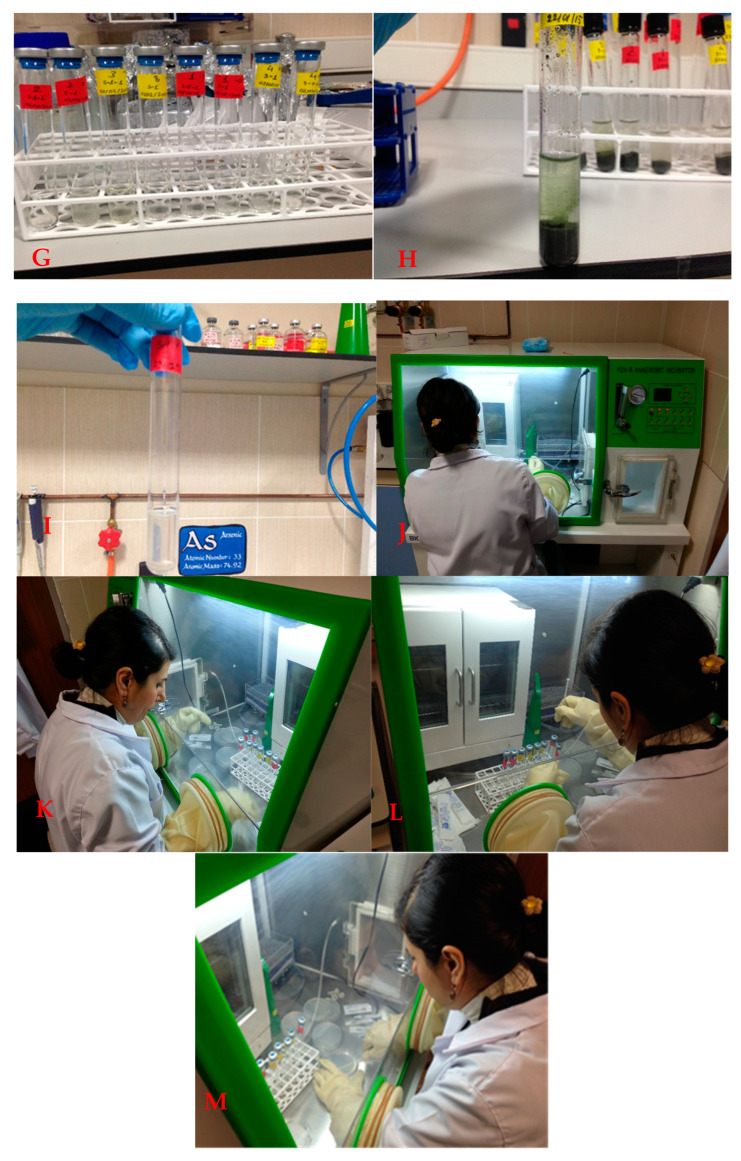

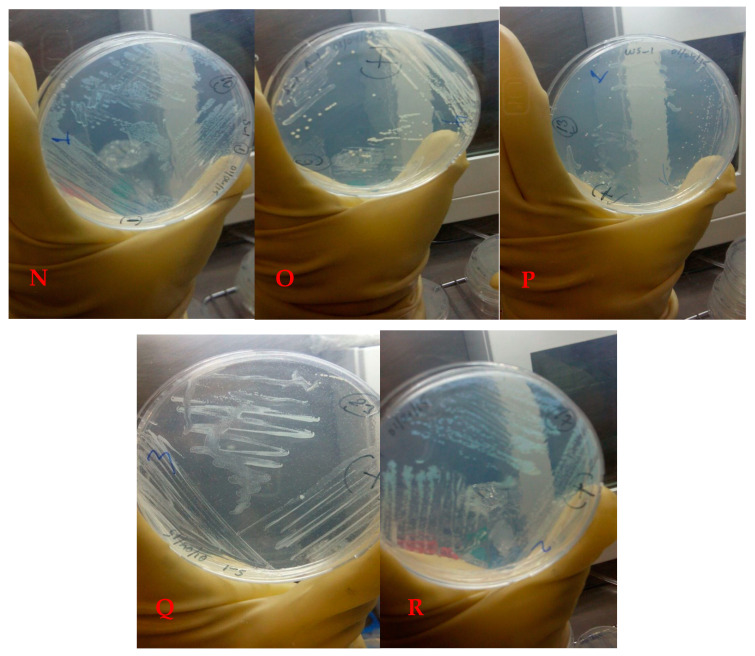
Isolation of anaerobic and alkalifilic arsenate-reducing and arsenite-oxidizing bacteria. Preparation of media with Hungate technique (**A**–**E**), Bacterial growth in Balch and Hungate tubes (**F**–**I**), Obtaining pure culture using anaerobic chamber (**J**–**M**), Different types of colonies formed on agar media (**N**–**R**).

All isolation and purification assays were carried out according to Widdel et al. [26]. Saltikov et al. [27], Zargar et al. [12] and Sarkar et al. [28]. Anaerobic conditions were achieved by injecting nitrogen gas into the chamber with the Hungate Technique. Inoculation was carried out from both sediment and water samples to four different media. For the first inoculation, 5 mL of Big Soda Minimal Medium (BSM) [NaCl (20.0 g/L), KH_2_PO_4_ (0.48 g/L), K_2_HPO_4_ (0.6 g/L), (NH_4_)2SO_4_ (0.45 g/L), MgSO_4_ (0.20 g/L), 2 mg/mL vitamin B_12_ (200 µL/L), SL10 trace element solution (2 mL/L) and 1000× vitamin solution (20 mL/L)] was used in Hungate tubes. A 5% percentage of 20× carbs solution [Na_2_CO_3_ (5.3 g/100 mL), NaHCO_3_ (2.1 g/100 mL)] was used to adjust 9.5 the pH of the medium. This medium was a modified one in terms of NaCl amount. Because the NaCl content of Van Lake was 20‰. So, 20‰ NaCl was added instead of 50‰. Tubes containing BSM, 10 mM sodium arsenate [Na_2_HAsO_4_.7H_2_O] as an electron acceptor, and 10 mM Na-lactate (medium 1) or Na-acetate (medium 2) as electron donors were inoculated to select for arsenate [As(V)] reducing bacteria. For isolation of arsenite [As(III)] oxidizing bacteria, tubes containing BSM, 10 mM Na-nitrate as an electron donor, and 1 mM sodium arsenite (NaAsO_2_) (medium 3) or 0.5 mM NaAsO_2_ (medium 4) were used. Sediment sample (1 g) and water sample 8 (1 mL) were inoculated into medium 1–4 under anaerobic conditions. Samples were then incubated at 30 °C for 5 days. 1 mL of inoculum belonging to each tube was transferred to the same medium in Balch tubes and incubated at the same condition [12,26,27,28]. The transfer to Balch tubes was repeated three times. In the anaerobic chamber, the inoculum obtained from the last tube was transferred to BSM agar plates. In that step, from which tube the inoculum was taken, it was transferred to the same content of agar medium. After the growth was observed during the anaerobic incubation period (5 days), the colonies were patched on fresh BSM agar plates. Several morphologically different colonies were selected (see Appendix A Figure A2). For all arsenic-resistant isolates, glycerol stocks were prepared with a 25% final glycerol concentration and stored at −80 °C.

### 2.5. Arsenic Transformation Assay

An arsenic transformation assay was performed using a silver nitrate test [28]. Different kinds of agar plates (Luria Bertani (LB), BSM, and Chemically Defined Medium [CDM]) were supplemented with sodium arsenate (10 mM) and sodium arsenite. All isolates were streaked onto agar plates and incubated at 30 °C for 5 days. After the incubation period, all plates were flooded with a 0.1 M silver nitrate (AgNO_3_) solution conserved in dark conditions. Color precipitations were examined to evaluate the results. A brownish and a bright yellow precipitate indicated the presence of silver arsenate (Ag_3_AsO_4_) (arsenite oxidizing bacteria) and silver arsenite (Ag_3_AsO_3_) (arsenate reducing bacteria), respectively. As positive controls, *Shewanella* ANA-3 [27] and *Halomonas* sp. BSL-1 [29] were used.

### 2.6. Physiological Characterization and Identification of Arsenic-Resistant Bacteria

Arsenic-resistant bacteria obtained from different water and sediment samples of Lake Van were initially characterized in terms of colony morphologies (color, shape, size) and basic microscopic observations (Gram, spore, capsule, and PHB stains) [13]. Isolates were then biochemically analyzed for the activities of nitrate reduction [30], starch hydrolysis, gelatin liquefaction test [31], production of extracellular lipase, lecithinase [32], protease [31], carbohydrate assimilation, along with fermentation tests like glucose, lactose, mannose, fructose, and galactose [30,33].

For molecular identification, the genomic DNA of arsenic-resistant isolates was obtained with PureLink^®^ Genomic DNA Kits according to the manufacturer’s instructions. For checking the integrity of DNA, gel electrophoresis was performed using 1% agarose gel. The universal primers used for the isolation of the 16S rRNA gene region were 11F (5′-AGTTGATCCTGGCTCAG-3′) and 1492R (5′-ACCTTGTTACGACTT-3′) [12]. The reaction mixtures were composed of 5 µL 10X PCR buffer, 2.5 mM dNTPs, 10 pmol each primer, 10 ng DNA template, 3 units Taq DNA polymerase, and sterile deionized water to a final volume of 50 µL. The PCR protocol for amplification of the 16S rRNA gene was comprised of denaturation at 94 °C for 5 min, followed by 35 cycles at 94 °C for 1 min, annealing at 48 °C for 45 s, and extention at 72 °C for 2 min. A final extention was carried out for 10 min at 72 °C. To reveal individual differences, amplified 16S rRNA gene regions of each isolate were cut with *Eco*RI (CutSmart Biolabs, New England Biolabs, Ipswich, MA, USA, R3101S), *Rsa*I (CutSmart Biolabs, New England Biolabs, R0167S), and *Hae*III (CutSmart Biolabs, New England Biolabs, R01108S) restriction enzymes according to manufacturer’s instructions. The bidirectional sequence analysis of purified amplicons (with Sephadex^®^ G-50 kit, Sigma Aldrich G5080, Merck KGaA, Darmstadt, Germany) was conducted with ABI 3130xl genetic analyzer (Applied Biosystems, Waltham, MA, USA) using the same primers. Chromatograms were analyzed with Finch TV, ApE, and Bioedit programs. 16S rRNA partial sequence of selected isolates was compared to reference sequences in the GenBank database using BLASTN, and accession numbers for the strains were obtained.

### 2.7. PCR Amplification of Arsenic-Related Marker Genes

In this study, we examined some putative arsenic resistance genes, including cytosolic arsenate reductase gene (*ars*C) [10,28], arsenite efflux gene (*ars*B) [10], and respiratory arsenate reductase gene (*arr*B) [34]. PCR amplification of *ars*C, *ars*B, and *arr*B gene regions was done by using arsC-1-F (5′-GTAATACGCTGGAGATGATCCG-3′) and *ars*C-1-R (5′-TTTTCCTGCTTCATCAACGAC-3′), *ars*B-1-F (5′-CGGTGGTGTGGAATATTGTC-3′) and *ars*B-1-R (5′-GTCAGAATAAGAGCCGCACC-3′), and *arr*B-F (5′-AACACGAACGACGGTATTCACTGG-3′) and *arr*B-R (5′-ATACCTTGCTCTGTGGATCATCTA-3′) primers, respectively. The reaction mixtures composed of 2.5 µL 10X PCR buffer, 2.5 mM dNTPs, 10 pmol each primer, 10 ng DNA template, 2 units Taq DNA polymerase, 5% DMSO, 2 mM MgCl_2_ and sterile deionized water to a final volume of 25 µL for *ars*C and *ars*B gene regions. The same mixture was used to amplify the *arr*B gene region, excluding the amount of template DNA and primers. A total of 25 ng DNA and 20 pmol of each primer were optimized for amplification. PCR profile was as follows: initial denaturation at 94 °C for 3 min (5 min for *arr*B), followed by 30 cycles (for *ars*C and *ars*B) or 35 cycles (for *arr*B) of denaturation (94 °C, 30 s for *ars*C and *ars*B; 40 s for *arr*B), annealing (56 °C, 30 s for *ars*C and *ars*B; 62 °C 1 min for *arr*B), extension (72 °C 30 s for *ars*C and *ars*B, 1 min for *arr*B), and final extention at 72 °C for 7 min. Obtaining 370 bp, 219 bp, and 500 bp bands as amplification products for *ars*C, *ars*B, and *arr*B was evaluated as having positive results.

### 2.8. Effect of Physicochemical Parameters

Hydrogen ion concentration (pH), temperature, and salinity variation were selected as physicochemical parameters for bacterial growth. All assays were performed using the microplate technique in LB broth with 2% NaCl or pH 9.5. The amount of bacterial inoculum was designated as 1 McFarland. Each arsenic-resistant strain was inoculated into three microplate wells for all different physical parameters under anaerobic conditions. A 5-day incubation period was preferred for all physicochemical parameters. 25 °C, 30 °C, 35 °C, 45 °C, 55 °C, and 65 °C were selected for incubation temperature. Depending upon the optimum temperature, they were incubated in microplates with different pHs of 4.5, 5.5, 6.5, 7.5, 8.5, 9.5, and 10.5 to determine the optimum pH. To adjust the pH, 0.1 M different buffers (acetate, phosphate, and carbonate) were used. Similarly, the effect of salinity variation on bacterial growth was studied in a microplate. For this assay, LB with pH 9.5 was amended with different concentrations of NaCl as 1%, 2%, 3%, 4%, 5%, 6%, 7%, 8%, 9%, 10%, and 11%. After an anaerobic 5 days incubation period, the optical density of the growing cultures in different conditions was determined spectrophotometrically at 620 nm [13]. An amount of 20 µL of 2,3,5 triphenyl tetrazolium chloride (TTC) with 10% concentration for each microplate well was used as the indicator of viability for clear observation of bacterial viability. After adding TTC, all microplates were incubated under anaerobic conditions for an additional day. The formation of red-colored triphenyl formazan (TPF) was evaluated as a positive result [35].

### 2.9. Arsenic Tolerance Assay

The resistance of the strains against As(V) [Na_2_HAsO_4_.7H_2_O] and As(III) (NaAsO_2_) was determined by minimum inhibitory concentration (MIC). To screen the strains based on arsenic resistivity, the overnight grown culture was inoculated in a microplate well containing LB with 2% NaCl and pH 9.5, As(V) or As(III) as sources of the arsenic. All concentrations of arsenic were examined in three wells at 30 °C. The MIC value was tested by growing arsenic metabolizing bacteria in different concentrations of As(V) (0–320 mM) and As(III) (0–32 mM) [28]. The bacterial growth was determined via optical density measurement at 620 nm, and vitality was corrected by using TTC. The MIC was defined as the lowest concentration of As(V) and As(III) that suppressed bacterial growth [35].

## 3. Results

### 3.1. Sample Collection

To isolate arsenic-resistant anaerobic bacteria, sediment and water samples of Lake Van were collected seasonally. For each season, the same sampling coordinate was used. After sampling, 4 different sediment and water samples were obtained. The pH of the samples was found to be between 9.52–9.86. As expected, temperature, dissolved O_2,_ and moisture changed for each season (Table 1).

### 3.2. Determination of Heavy Metal Amount in Van Lake Samples

According to EPA and WHO, 10 ppb (µg/L) has been indicated as the limit of arsenic concentration.

The results of 24 different heavy metal analyzes of sediment and lake water samples are shown in Table 2. In general terms, as expected, it was determined that the amounts changed depending on seasonal changes (Table 2).

**Figure A3:**
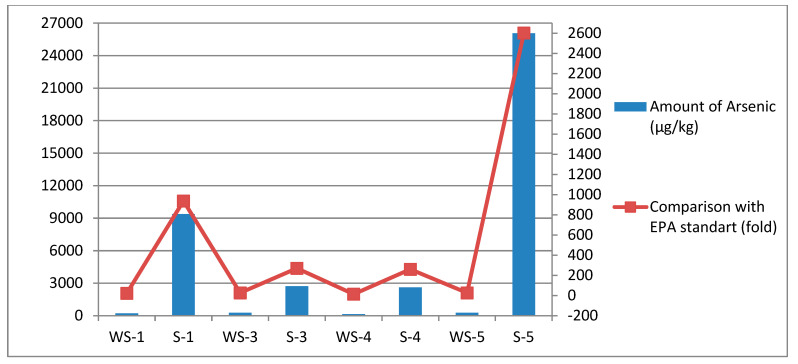
Comparison of arsenic amounts of Lake Van samples.

When evaluated in terms of arsenic, it was determined to be above the standards, especially in the sediment samples. Within the scope of the study, seasonal sampling was carried out with the thought that seasonal differences may change the amount of arsenic in the lake water and sediment and therefore change the dominant microbiota. The ICP-MS analysis showed that the total amount of arsenic in the sediment was very high in the autumn season, and there was a gradual decrease in the winter, spring, and summer seasons. It was determined that the total amount of arsenic found in the sediment, especially in the autumn season, resulted in a 10-fold decrease in the summer (see Appendix A, Figure A3).

### 3.3. Isolation of Arsenate and Arsenite Metabolizing Anaerobic Bacteria

A total of 81 arsenic-resistant bacteria were isolated from the samples of lake water and sediment from Lake Van. After this stage, the studies were continued with the 12 arsenic-resistant isolates selected. They were designated as 4-S-1-1 A, 4-S-1 A2, 1-WS-1, 1-WS-1-1, 1-S-1 (2), 2-WS-1-1, 3-S-1 K, 3-S-1 A, 1-S-3-1, 3-S-4-1, 1-WS-5-1, and 1-S-5 (3) and also they were isolated in different season and different media content (Table 3).

### 3.4. Silver Nitrate Test

The AgNO_3_ screening technique was used to detect the oxidation of As(III) to As(V) or the reduction of As(V) to As(III) [35]. Although different media and conditions were used in addition to the media specified in the literature within the scope of this experiment, no results were obtained for the alkaliphilic isolates. 

### 3.5. Identification of Arsenic-Resistant Bacteria

It was determined that 12 isolates selected within the scope of the study belong to eight different genera (Table 3). According to the results of partial 16S rRNA sequence analysis, it was determined that there were similarities with various species at rates ranging from 98% to 100%. Molecular identification of 7 strains could be made at genus level and did not show similarity with any other species within the ranges suitable for the criteria. It has been determined that 4 strains (3-S-1 K, 3-S-4-1, 1-WS-5-1, and 1-S-5 (Table 3)) are members of the *Halomonas* genus, which is also found extensively in other soda lakes in the world. It was observed that 2 strains (1-S-1 (2) and 1-S-3-1) in the sediment samples had members of *Anaerobacillus*, an obligate anaerobic species [36].

When we look at the determined characteristics of arsenic-resistant strains, it is seen that they exhibit different profiles depending on the strain difference. As a common feature of all strains, it appears there is no extracellular gelatinase activity (Table 4).

### 3.6. Detection of Arsenic Marker Genes

Within the scope of the study, it was investigated whether microorganisms have certain gene regions for respiration of As(V) and are resistant to arsenic. When strains are evaluated in terms of having *ars*C, *ars*B, and *arr*B gene regions selected as target gene regions, it has been determined that all strains have shown a common profile in terms of gene regions and all species have the *ars*C gene, which is the arsenate reductase enzyme gene, which confers the ability of microorganisms to convert arsenate to arsenite prior to extrusion of the latter oxidation [37]. In the meantime, it was observed that none of the strains had the *ars*B and *arr*B gene regions, although the required products (219 bp and 500 bp products for *ars*B and *arr*B genes, respectively) were observed in the positive controls. The products of the gene regions were not observed in the isolates.

### 3.7. Effect of Physicochemical Parameters

**Figure 2:**
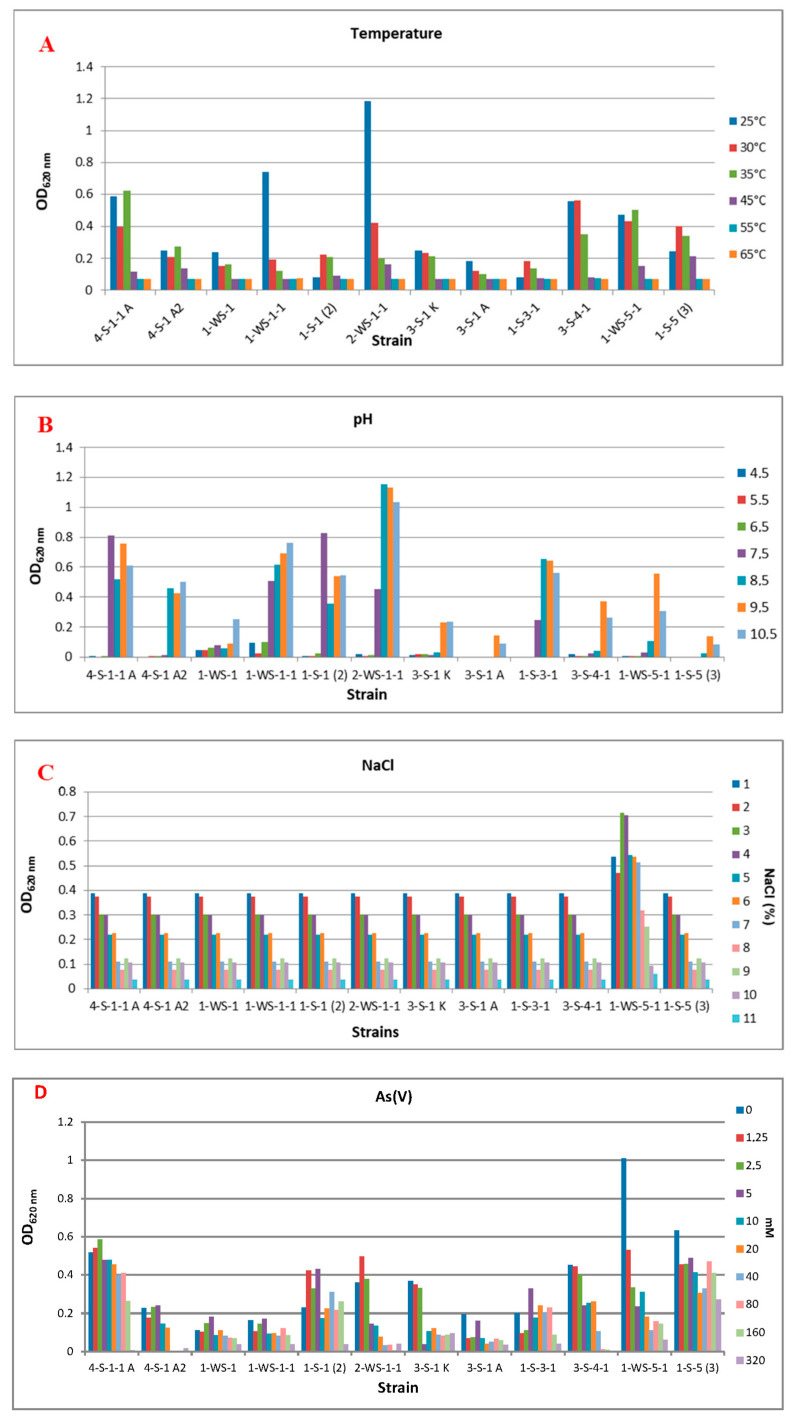

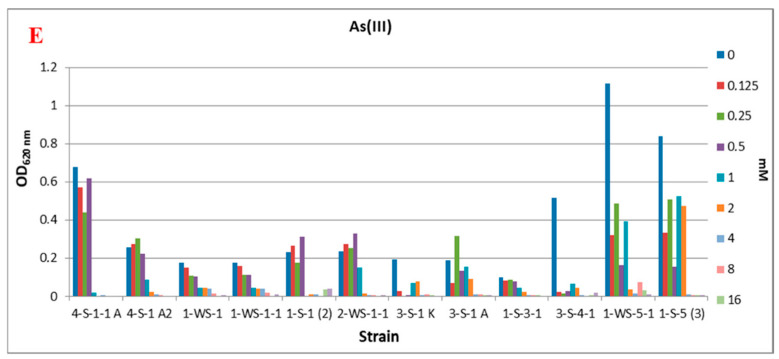
Effect of some physicochemical parameters and different concentrations of As(V) and As(III) on bacterial growth. (**A**) Temperature, (**B**) pH, (**C**) NaCl, (**D**) As(V), (**E**) As(III).

As a result of the experiment carried out under anaerobic conditions, it was determined that there was no microbial activity at 55 °C and 65 °C in common, but there were differences at other temperatures. It was observed that the temperatures at which the best reproduction took place were 25 °C and 30 °C. It was determined that strain 2-WS-1-1 (*Nitrincola* sp.) was the strain that showed the best growth at these temperatures. By increasing the incubation temperature to 45 °C, it was observed that strain 1-S-5 (3) (*Halomonas* sp.) exhibited microbial activity, unlike other strains (Figure 2A).

It was determined that there was no growth at pH 4.5, 5.5, and 6.5 as a common feature in all strains, and strain 1-S-1 (2) (*Anaerobacillus* sp.) showed intense growth, unlike the others. While it was determined that strains 3-S-1 A (*Marinobacter halophilus*), 3-S-4-1 (*Halomonas* sp.), 1-WS-5-1 (*Halomonas campisalis*), and 1-S-5 (3) (*Halomonas* sp.) at pH 9.5 showed higher microbial activity than other strains, at pH 10.5, 4-S-1 A2 (*Idiomarina* sp.), 1-WS-1 (*Branchybacterium paraconglomeratum*), and 1-WS-1-1 (*Microbacterium schleiferi*) strains showed better growth under these conditions (Figure 2B).

When the microbial activity is compared depending on the changes in the salt concentration in the medium, it has been determined that all strains can grow in environments containing 1–11% NaCl, with the concentration at which they grow best (Figure 2C).

### 3.8. MICs

Strains 4-S-1-1 A (*Alkalimonas delamerensis*) and 3-S-4-1 (*Halomonas* sp.) did not show any significant microbial activity in the medium containing 320 mM As(V). The strain 1-S-5 (3) (*Halomonas* sp.) showed good growth in the medium containing 160 mM and 320 mM As(V), unlike the other strains. In the medium that does not contain any As(V), it was determined that the strain that showed the best growth was strain 1-WS-5-1 (*Halomonas campisalis*). When we look at the As(V) concentrations that can be tolerated as a result of incubation under anaerobic conditions, it is seen that growth is generally high at values between 0–5 mM (Figure 2D).

In the environment that does not contain As(III), it was determined that strains 4-S-1-1 A (*Alkalimonas delamerensis*), 3-S-4-1 (*Halomonas* sp.), 1-WS-5-1 (*Halomonas campisalis*) and 1-S-5 (3) (*Halomonas* sp.) showed significantly better growth. It was observed that As(III) concentrations higher than 1 mM in the medium were generally not tolerated by the strains, and only strain 1-S-5 (3) (*Halomonas* sp.) could grow under these conditions (Figure 2E).

## 4. Discussion

The amount of water in deep terminal lakes such as Lake Van may change seasonally [22,23]. In this context, samplings were carried out to represent 4 seasons, with the thought that seasonal differences may change the amount of arsenic in the lake water and sediment and therefore change the dominant microbiota. While it is expected that the warming of the weather will decrease the amount of lake water and increase the amount of arsenic, it has been determined that there is approximately a similar amount of arsenic in the spring (2.730 µg/L) and summer (2.61 µg/L) seasons for sediment samples. However, in the transition to autumn (26.070 µg/L), it was determined that there was 10 times more arsenic than the amount of arsenic obtained from the spring and summer seasons. When the amounts of arsenic in the summer (2.61 µg/L) and spring (2.730 µg/L) seasons are compared with the winter data (9.370 µg/L), it has been determined that it contains three times more arsenic in winter. However, it has been revealed that there is a threefold decrease in the amount of arsenic in the transition from autumn (26.070 µg/L) to winter (9.370 µg/L). Although no such data has been encountered regarding Lake Van, as a result of the study conducted by Savarimuthu et al. in 2006, it was determined that the amount of arsenic in the water wells of West Bengal, India, changes seasonally. The lowest value is in summer (694 µg/L, 906 µg/L, and 794 µg/L for summer, monsoon, and winter, respectively) [38]. Because the sampling area is a region where anthropogenic inputs are quite high, it is thought that the increase is considered high due to the accumulation of arsenic in the sediment in the next season as a result of the intense activity in the summer months, because the sampling point is a region with a high potential for anthropogenic pollution due to various water sports activities and the presence of various businesses in its vicinity. As an indicator of this, it has been shown some regions are unsuitable for microbiological criteria in the Van-Edremit region [21]. The fact that the amount of arsenic contained in the lake water samples belonging to different seasons was approximately the same (0.214 µg/L, 0.263 µg/L, 0.14 µg/L, 0.261 µg/L for winter, spring, summer, and autumn, respectively), was considered as a data supporting this situation.

Isolation and identification studies were carried out in various lakes with characteristics similar to Lake Van. Alkaline and salty lakes, such as Lake Van, are potential isolation sources for arsenic-resistant bacteria, such as Mono Lake, Searles Lake, and Dali Lake. The obtained data is important because no studies have been carried out at Lake Van in this context, and it is the largest soda lake in the World [20]. Arsenic-resistant bacteria have been isolated from lakes such as Searles Lake and Mono Lake in the United States, which have similar characteristics to Lake Van, and have been introduced to the literature as a new species. It is seen that similar studies were carried out at Dali Lake in China [14,39,40]. When culture-dependent and metagenomic studies conducted with other lakes that have geochemically similar characteristics to Lake Van are examined, it is seen that Gammaproteobacteria members are dominant in such soda lakes [41]. As a matter of fact, as a result of the study, Gammaproteobacteria members such as *Halomonas*, *Alkalimonas*, *Idiomarina*, *Nitrincola*, and *Marinobacter* were found to be lake biota members (Table 3). Apart from Gammaproteobacteria members, it has been shown in studies based on various 16S gene sequences that Firmicutes, Actinobacteria, and Bacteriodetes members dominate in alkaliphilic lakes in general [41]. Since our study aimed to detect arsenic metabolizing, alkaliphilic, and culturable bacteria, the identified species were limited. However, it has been determined that the bacteria living in Lake Van with this feature are members of Gammaproteobacteria (*Halomonas*, *Alkalimonas*, *Idiomarina*, *Nitrincola*, and *Marinobacter*), Actinobacteria (*Branchybacterium paraconglomeratum* and *Microbacterium schleiferi*) and Firmicutes (*Anaerobacillus* sp.) in accordance with the literature (Table 3). It is important and necessary to detect microorganisms living in habitats, such as Lake Van, where extreme conditions dominate, using cultural and culture-independent techniques. Because these microorganisms are the source of many biotechnological products, they are also important in terms of understanding the various cycles prevailing in the lake ecosystem due to their important role in the food chains. As a result of the dominant species in the cycles, such as carbon, nitrogen, and phosphorus and their metabolic activities, the products released are very important in terms of the quality of the water, the determination of the species that can live in these conditions, and especially the preservation of the existence of various endemic creatures.

The most biotechnological use of arsenic-resistant bacteria is the removal of arsenic pollution, a major environmental and public health problem. As(V) reduction and As(III) oxidation processes can potentially promote arsenic removal from contaminated areas. Because both processes directly affect the mobility and bioavailability of As. As a result of this, microbial activities play a key role in biogeochemical arsenic cycling [42]. Therefore, arsenic metabolism pathways should be determined to use suitable strains in the assays. In this context, the AgNO_3_ screening technique is highly preferred due to its low cost [28]. However, in this study, although different environments and conditions were used in addition to the experimental environments specified in the literature, no results could be obtained for alkaliphilic isolates. These assays have determined that the silver nitrate test has the disadvantages of being very toxic and, at the same time, is unable to give results in every environment and organism for the determination of arsenate reductase and arsenite oxidase enzyme activities.

Another way to identify pathways is to screen for resistance genes. In the study’s results, it was observed that no strains possessed the arsB gene region, an integral membrane protein that can extrude arsenite from the cell cytoplasm, thus reducing arsenite accumulation [43]. A novel gene cluster encoding respiratory As(V) reductase has been identified for the first time in *Shewanella* ANA-3. There are two genes in this gene cluster, *arr*A and *arr*B [44]. Within the scope of our study, the *arr*B gene region was selected as the target region, but it was determined that arsenic-resistant strains did not have this gene region. However, a 500 bp product was obtained, which indicates the positive result in the *Shewanella* ANA-3 strain used as a positive control. Various microorganisms capable of metabolizing various toxic forms of arsenic exist in nature. As a result of the evolution of each species, in accordance with the habitat conditions it is adapted to, they have the appropriate gene regions and, therefore, their products. In this context, within the scope of our study, strains belonging to different genera and species, which also have different Gram reactions and show variations in oxygen requirements, were obtained from various water and sediment samples of Lake Van. However, it was determined that only the *ars*C gene region was found in all strains from the gene regions examined in general. While it is expected that the strains identified as *Anaerobacillus* sp. from the obtained strains are anaerobic in terms of oxygen requirement, it is expected to contain the *arr*B gene region, which is one of the respiratory arsenate reductase genes, but the opposite result was obtained. Further studies should be conducted to determine whether it is a new species and whether it has a new resistance gene that functions under anaerobic conditions.

Electron donors in the environment are also of great importance in the metabolism of various forms of arsenic [45]. As a result of the experiment, similar to other studies [46], it was determined that the obtained arsenic-resistant strains preferred Na-lactate as an electron donor in environments containing As(V).

Considering the As(V) and As(III) tolerances of the strains, although As(III) is a more toxic form, it has been determined that it can reproduce in environments containing As(III). In particular, in the *Halomonas* sp. 1-S-5 (3) strain obtained from the autumn season sediment sample, where the total arsenic content is the highest, growth was intense in the medium containing As(III), and the MIC value increased to 2 mM. In the presence of As(V) in the medium, it is seen that the MIC value for this strain reached >320 mM. Although this strain was isolated in a medium containing As(V), it was determined that it was resistant to both As(V) and As(III). A similar situation was observed in *Anaerobacillus* sp. 1-S-1 (2) and 1-S-3-1 strains. When the studies are examined, *Idiomarina* sp. [47], *Microbacterium* schleiferi [48], *Anaerobacillus* sp. [49], *Nitrincola* sp. [50], *Bacillus* sp., and *Halomonas* sp. [51] data on arsenic resistance and resistance genes are seen. However, no data were found for *Alkalimonas delamerensis* [52], *Barnchybacterium paraconglomeratum* [53], and *Marinobacter halophilus* [54] strains and presented as preliminary data.

## 5. Conclusions

As an epilog, it should be emphasized that Lake Van is the largest soda lake in the world, and there are no studies carried out in Turkey or the world regarding anaerobic arsenic-metabolizing bacteria. With the increasing global warming and anthropogenic pollution, arsenic-contaminated areas are increasing. As a result, the increase in arsenic exposure brings about serious health problems and also causes an increase in resistance to various antimicrobial agents. These results make the arsenic problem one of the most important global problems since millions of people worldwide are constantly and regularly exposed to arsenic. When evaluated in terms of Lake Van, the increase in arsenic accumulation in the lake water and, therefore, in pearl mullet, an endemic fish species consumed as food by the local people, causes public health and the continuation of this species to be endangered. Due to the use of lake water for irrigation purposes in agricultural areas, the size of the threat is increasing. Therefore, isolation and identification of resistant strains from environments with high arsenic contamination, as well as identifying the pathways they use to metabolize arsenic, have become essential. This is because the strains obtained can be used for the microbial remediation of arsenic, and at the same time, information about the microbiota in these extreme environments can be obtained, and possible new genus and species can be brought to the literature. At the same time, a world arsenic map and statistics were obtained by determining the amount of arsenic in isolation areas. The data herein will contribute to the sustainability of Lake Van and the understanding of bacterial arsenic metabolism with the contribution of different strains. The study will be evaluated as a model organism source for arsenic bioremediation studies and for being the first data on Lake Van, the world’s largest soda lake.

## Figures and Tables

**Table 1:** Various parameters of seasonal water and sediment samplings.

Season	Winter	Spring	Summer	Autumn
Sampling date	20 January 2015	12 May 2015	28 August 2015	25 November 2015
Sample code				
Water	**WS-1**	**WS-3**	**WS-4**	**WS-5**
Sediment	**S-1**	**S-3**	**S-4**	**S-5**
Coordinate *	38°25′31″ N 43°16′53″ E	38°25′31″ N 43°16′53″ E	38°25′31″ N 43°16′53″ E	38°25′31″ N 43°16′53″ E
Hour *	14:32	09:55	10:30	14:45
pH **	9.52	9.86	9.82	9.80
Temperature **	3 °C	10.8 °C	23.2 °C	11.1 °C
Dissolved O_2_ **	11.31 mg/L	9.86 mg/L	6.39 mg/L	8.82 mg/L
Moisture *	87%	91%	37%	69%
Pressure *	811 hPa	834 hPa	1021 hPa	815 hPa

*: iGPS was used. **: HQ40d multi LDO101 and HQ40d multi PHC101 were used.

**Table 2:** Concentration of some heavy metal for Lake Van samples for each season.

Heavy Metal	Season								
	Winter		Spring		Summer		Autumn		
	WS-1	S-1	WS-3	S-3	WS-4	S-4	WS-5	S-5	
Na	7587.000	17,430.000	7230.000	4885.000	8471.00	6616.0	13,690.000	20,800.00	Concentration of heavy metal (mg/kg or mg/L)
Mg	104.000	19,660.000	98.060	7028.000	107.60	7716.0	167.200	73,670.00	
Al	0.253	21,100.000	0.005	7931.000	1.40	8848.0	4.032	24,830.00	
K	409.200	7220.000	462.000	3401.000	459.10	2774.0	737.400	11,360.00	
Ca	34.060	125,200.000	3.949	30,760.000	11.20	25,980.0	23.080	233,500.00	
V	0.005	57.130	0.004	22.800	0.01	19.58	0.019	79.570	
Cr	0.012	80.760	ND	30.950	0.01	30.03	0.083	131.800	
Mn	0.024	509.900	ND	173.400	0.04	148.70	0.057	771.300	
Fe	0.080	18,800.000	ND	7043.000	3.06	7272.0	2.491	281,800.00	
Co	ND	10.520	ND	3.963	ND	3.8	ND	16.010	
Ni	0.011	69.120	0.021	26.540	ND	31.4	0.036	93.030	
Cu	ND	14.230	ND	7.818	ND	4.9	0.185	8.558	
Zn	0.714	53.840	0.250	31.030	ND	13.13	0.222	26.100	
As	0.214	9.370	0.263	2.730	0.14	2.61	0.261	26.070	
Se	0.150	0.999	0.100	4.830	0.07	2.49	0.039	1.207	
Mo	0.012	1.423	0.007	1.727	0.02	1.01	0.027	1.194	
Ag	ND	81.610	ND	0.235	ND	ND	0.008	0.354	
Cd	ND	0.186	ND	0.085	ND	0.06	ND	0.199	
Sn	ND	1.711	ND	0.740	0.07	0.29	0.120	6.546	
Sb	ND	0.299	ND	0.164	ND	ND	ND	0.528	
Ba	0.003	133.800	0.013	56.880	0.04	38.78	0.043	297.200	
Hg	ND	ND	ND	0.591	0.13	0.015	ND	ND	
Tl	ND	0.259	ND	0.103	ND	ND	ND	0.181	
Pb	0.045	8.456	ND	4.075	0.003	2.35	0.024	15.160	

ND: Not determined. Those beginning with the WS and S codes represent samples of lake water and sediment, respectively.

**Table 3:** The features of isolation source of arsenic-resistant bacteria and the results of 16S rRNA analysis of the isolates.

No.	Isolate	Sample	Season	Medium		Closest Neighbor	Percentage Similarity	Accession Number
				e^−^ acceptor	e^−^ donor			
1	4-S-1-1 A	Sediment	Winter	Na-nitrate (10 mM)	As(III) (0.5 mM)	*Alkalimonas delamerensis*	98%	KY681793
2	4-S-1 A2	Sediment				*Idiomarina* sp.	99%	KY681796
3	1-WS-1	Lake water		As(V) (10 mM)	Na-lactate (10 mM)	*Branchybacterium paraconglomeratum*	99%	KY681785
4	1-WS-1-1	Lake water				*Microbacterium schleiferi*	99%	KY681786
5	1-S-1 (2)	Sediment				*Anaerobacillus* sp.	99%	KY681780
6	2-WS-1-1	Lake water		As(V) (10 mM)	Na-acetate (10 mM)	*Nitrincola* sp.	98%	KY989222
7	3-S-1 K	Sediment		Na-nitrate (10 mM)	As(III) (1 mM)	*Halomonas* sp.	99%	KY681792
8	3-S-1 A	Sediment				*Marinobacter halophilus*	99%	KY681791
9	1-S-3-1	Sediment	Spring	As(V) (10 mM)	Na-lactate (10 mM)	*Anaerobacillus* sp.	99%	KY681781
10	3-S-4-1	Sediment	Autumn	Na-nitrate (10 mM)	As(III) (1 mM)	*Halomonas* sp.	100%	KY989223
11	1-WS-5-1	Lake water	Summer	As(V) (10 mM)	Na-lactate (10 mM)	*Halomonas campisalis*	100%	KY681788
12	1-S-5 (3)	Sediment	Summer			*Halomonas* sp.	99%	KY681784

**Table 4:** Some characteristics of arsenic-resistant bacteria from Lake Van.

Characteristics	Strains											
	1	2	3	4	5	6	7	8	9	10	11	12
Colony color	Blue	Blue	Cream	Cream	Cream	Cream	Cream	Blue	Transparent	Cream	Cream	Blue
Gram staining	Gr(-)	Gr(-)	Gr(+)	Gr(+)	Gr(+)	Gr(-)	Gr(-)	Gr(-)	Gr(+)	Gr(-)	Gr(-)	Gr(-)
Cell morphology	Bacil	Bacil	Bacil	Bacil	Bacil	Bacil	Bacil	Bacil	Bacil	Bacil	Bacil	Coccobacil
Formation of												
Capsule	-	+	+	+	+	+	+	+	+	+	+	+
Endospore	-	+	+	-	+	+	+	-	-	+	+	+
PHB	+	+	+	-	+	+	+	-	-	-	-	+
Nitrate reduction	+	-	-	-	+	+	-	+	+	+	+	+
Production of												
Lipase	-	-	-	-	-	-	-	-	+	-	-	-
Lecithinase	-	-	-	-	-	+	+	-	-	+	-	+
Amylase	+	-	+	-	+	-	-	-	+	+	+	+
Protease	-	+	-	-	+	+	+	+	-	-	-	-
Gelatinase	-	-	-	-	-	-	-	-	-	-	-	-
Assimilation of												
Glucose	+	+	+	+	+	+	+	+	+	+	+	+
Lactose	+	+	+	+	+	+	+	+	+	+	+	+
Mannose	+	+	-	+	+	+	+	+	+	+	+	+
Fructose	+	+	+	+	+	+	+	+	-	+	+	-
Galactose	+	+	+	+	+	+	-	-	-	+	+	+
Fermentation of												
Glucose	+	-	-	-	-	+	-	-	-	-	+	-
Lactose	+	-	+	+	-	+	+	+	-	+	+	+
Mannose	+	-	+	-	-	+	+	+	-	+	-	+
Fructose	+	+	-	-	-	+	-	-	-	+	+	+
Galactose	-	+	+	-	-	+	-	-	-	+	+	+

1: 4-S-1-1 A, 2: 4-S-1 A2, 3: 1-WS-1, 4: 1-WS-1-1, 5: 1-S-1 (2), 6: 2-WS-1-1, 7: 3-S-1 K, 8: 3-S-1 A, 9: 1-S-3-1, 10: 3-S-4-1, 11: 1-WS-5-1, 12: 1-S-5 (3). PHB: Poly-β-hyrdoxybutyrate.

## Data Availability

The data sets and materials supporting the results of this article are included within the article and also in Appendix A.
